# *PPP2R1A* Mutation: A Critical Amplifier for Immune Checkpoint Blockade Therapy Efficacy

**DOI:** 10.1002/mco2.70511

**Published:** 2025-11-20

**Authors:** Yuanzhuo Gu, Zhengkui Zhang, Fangfang Zhou

**Affiliations:** ^1^ Department of Gynecological Oncology Women's Hospital Zhejiang University School of Medicine Hangzhou China; ^2^ The First Affiliated Hospital the Institutes of Biology and Medical Sciences Suzhou Medical College Soochow University Suzhou Jiangsu China

1

In a recent paper published in *Nature* [1], Dai et al. revealed that *PPP2R1A* mutations in ovarian clear cell carcinoma (OCCC) are linked to superior survival following immune checkpoint blockade (ICB), driven by enhanced interferon (IFNγ) signaling and pre‐existing tertiary lymphoid structures (TLSs) that foster robust T‐cell immunity. This work nominates PPP2R1A as a promising therapeutic target to potentiate ICB efficacy across cancer types.

Despite the clinical impact of ICBs on various cancers, their efficacy in ovarian cancer remains limited, with even the most responsive OCCC subtype showing only a 15% response rate. This is underscored by the recent finding that durvalumab (anti‐PD‐L1) failed to improve progression‐free survival (PFS) or overall survival (OS) compared to chemotherapy in the MOCCA trial, despite some symptomatic improvement. Furthermore, traditional biomarkers, including PD‐L1 expression, tumor mutation burden (TMB), and *BRCA1/2* status, have proven inadequate in predicting ICB responses of OCCC, highlighting the pressing need to discover novel molecular determinants of response. The observed superiority of combination therapies over ICB monotherapy suggests a promising path forward. For instance, the combination of sintilimab (anti‐PD‐1) with bevacizumab (anti‐VEGF) significantly extended median PFS to 13.8 months versus 2.0 months with monotherapy [2], with a similar benefit observed for the nivolumab and ipilimumab combination in trial NCT04969887 of OCCC. This paradigm shift toward combinations underscores the need to identify which patients are most likely to benefit. In this context, the role of specific genetic alterations is gaining prominence. Beyond the established link between pathogenic *POLE/POLD1* mutations and high TMBs or *SETD2_Y1666* mutation, which modulate the tumor‐immune microenvironment [3], emerging evidence points to mutation‐specific responses. These collective findings illuminate two critical facets of OCCC immunotherapy: the necessity of rational combination strategies and the heterogeneity of treatment responses driven by distinct molecular features.

This study also initiated from the genomic fidelity of clinical cohort, according to 34 patients with platinum‐resistant OCCC receiving ICB therapy (NCT03026062 and NCT01928394), 11 cases (32.4%) were found to carry *PPP2R1A* gene mutations responded better in ICB treatment, predominantly at the R183 hotspot (81.8%). The mutations, encoding a scaffold subunit of protein phosphatase 2A (PP2A), were associated with a remarkable survival benefit and achieved a median OS of 66.9 months with ICB, seven times longer than that of the wild type group (9.2 months) (log‐rank *p* = 0.031). This effect was independent of cancer type (validated in pan‐cancer cohort) and co‐occurring mutations such as *ARID1A*, establishing *PPP2R1A* mutations as a highly specific and potent predictive biomarker for ICB, a finding that addresses a critical gap in OCCC precision immunotherapy (Figure 1).

**FIGURE 1:**
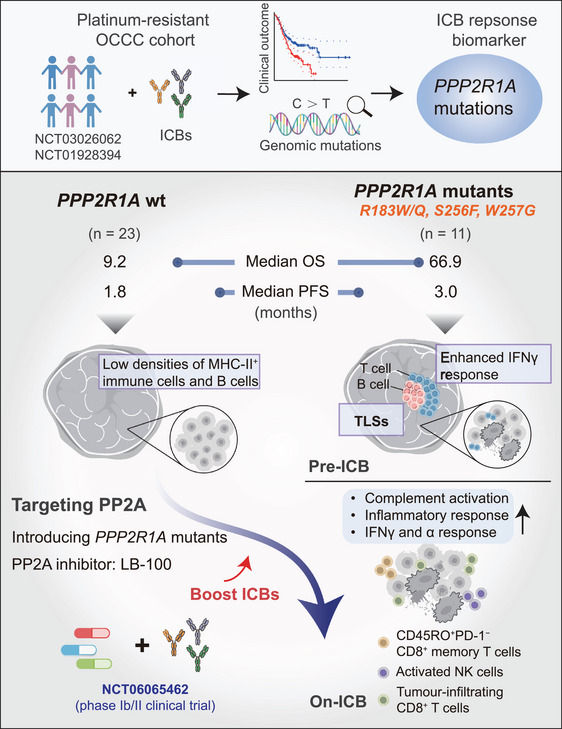
Cancer patients with *PPP2R1A* mutations benefit more from ICB treatment. Cancer patients with *PPP2R1A* mutations are associated with markedly prolonged OS and PFS following ICB treatment. The upper panel reveals how *PPP2R1A* mutation was identified, through two clinical trials (NCT03026062 and NCT01928394) of 34 patients with platinum‐resistant OCCC receiving ICB therapy. Clinical outcomes and genomic mutations were analyzed to identify the key biomarkers for ICB responses. The lower panel illustrates that the mechanisms of *PPP2R1A* mutations responded better in pre‐ and post‐ICB treatment. *PPP2R1A* mutant tumors exhibited enhanced IFNγ response, pre‐existing TLSs before ICB treatment, more immune infiltration, including CD8^+^ T cells and activated NK cells, and an expansion of CD45RO^+^CD8^+^ memory T cells post‐ICB. In contrast, *PPP2R1A* wild‐type tumors exhibited low densities of MHC‐II^+^ immune cells and B cells pre‐ICB treatment. Preclinical studies in vitro and in vivo suggest that targeting PP2A (a multimeric phosphatase with *PPP2R1A*‐ecoded scaffold) can boost ICB efficiency in tumors with wild‐type *PPP2R1A*, mainly through (1) knocking down or CRISPR‐editing wild‐type *PPP2R1A*, (2) introducing the mutant form, or (3) applying the PP2A inhibitor LB‐100. Currently, a Phase Ib/II clinical trial (NCT06065462) of ICB and LB‐100 combined therapy is conducting in OCCC patients without somatic *PPP2R1A* mutations.

Transcriptome (*n *= 29) and CO‐Detection by indEXing (CODEX) imaging (*n* = 28) demonstrated that PPP2R1A‐mutant tumors possess a pre‐existing, immunologically favorable microenvironment. Prior to ICB, these tumors exhibited IFNγ pathway enrichment, activated adaptive immune responses, higher densities of major histocompatibility complex (MHC)‐II^+^ immune cells and proliferative B cells (CD20^+^ Ki‐67^+^), and the presence of TLSs supporting B‐ and T‐cell activity. TLSs have been reported to be associated with good ICB response [4]. Post‐ICB, these patients showed further activation of key immune pathways (inflammatory response, complement pathway, and IFN‐α response), expansion of CD45RO^+^ CD8^+^ memory T cells and activated NK cells, along with heightened T cell receptor (TCR)/ B cell receptor (BCR) richness, indicating successful engagement and diversification of the immune response. In contrast, the wild‐type group lacked these features and failed to sustain responses.

The feasibility of targeting PPP2R1A to enhance immunotherapy was confirmed in preclinical models. Genetic perturbation of PPP2R1A (knockdown, mutation introduction, and CRISPR editing), or its pharmacological inhibition with LB‐100 [5], significantly sensitized tumor cells to immune attack. In vitro models revealed that the knockdown or mutation of *PPP2R1A* in tumor cells enhanced their sensitivity to killing by B7H3 chimeric antigen receptor T (CAR‐T) cells (with a 50% increase in apoptosis rate). In a humanized patient‐derived xenograft (PDX) model, mutant *PPP2R1A* tumors responded significantly to anti‐PD‐L1 therapy, with a 50% reduction in tumor weight (*p* < 0.0001). The PP2A inhibitor LB‐100 effectively mimicked the *PPP2R1A* mutation phenotype and synergized with ICB, supporting the launch of Phase Ib/II clinical trials (NCT06065462) to evaluate ICB combined with PP2A inhibition.

The study is limited by its sample size, which constrained the statistical power of subgroup analyses (e.g., only 4 AKT‐mutant cases), and the small number of PPP2R1A‐mutant cases in external validation cohorts (only four in the melanoma subgroup). These limitations hinder robust independent analyses and affect conclusion reliability. While PPP2R1A mutations are linked to an improved immune microenvironment (e.g., stronger IFNγ signaling and more T‐cell infiltration), the precise mechanisms by which PP2A function loss regulates immune pathways remain unclear. Additionally, the synergistic or independent interactions between *PPP2R1A* mutations and other known markers remain unknown, which is worth further exploration.

In summary, this paper reveals that the transformation of PPP2R1A from a mutational marker to a therapeutic target represents a paradigm shift in predicting and enhancing ICB efficacy, especially in OCCC. Key future directions include: (1) Elucidating the direct molecular mechanisms by which PP2A inhibition rewires tumor‐immune cell crosstalk, particularly its impact on T‐cell function and exhaustion; (2) Determining whether PPP2R1A mutation status can synergize with or act independently of existing biomarkers like TMB and PD‐L1 to refine patient stratification; (3) Validating its predictive power in large, prospective clinical trials across multiple cancer types. The ongoing clinical translation of PP2A inhibitors as “immune sensitizers” is promising, especially for wild‐type *PPP2R1A* patients in Phase Ib/II trials. This research trajectory holds significant potential to guide treatment for a broader population of cancer patients receiving immunotherapy, hoping it will bring therapeutic guidance to more cancer patients receiving or about to receive ICB treatment.

## Author Contributions

Y.G. conceived and drafted the manuscript. Y.G. and Z.Z. drew the figure. Z.Z. and F.Z. discussed and revised the manuscript. All authors have read and approved the article.

## Ethics Statement

The authors have nothing to report.

## Conflicts of Interest

The authors declare no conflicts of interest.

## Data Availability

The authors have nothing to report.
